# maldipickr dereplicates microbial MALDI-TOF spectra to facilitate multiplexed isolation

**DOI:** 10.1093/bioadv/vbag171

**Published:** 2026-06-17

**Authors:** Charlie Pauvert, David Wylensek, Selina Nüchtern, Thomas Clavel

**Affiliations:** Functional Microbiome Research Group, Institute of Medical Microbiology, University Hospital of RWTH Aachen, Aachen 52074, Germany; Functional Microbiome Research Group, Institute of Medical Microbiology, University Hospital of RWTH Aachen, Aachen 52074, Germany; Functional Microbiome Research Group, Institute of Medical Microbiology, University Hospital of RWTH Aachen, Aachen 52074, Germany; Functional Microbiome Research Group, Institute of Medical Microbiology, University Hospital of RWTH Aachen, Aachen 52074, Germany

## Abstract

**Motivation:**

Microbiologists use MALDI-TOF for fast and cheap identification of microbial isolates. However, most standard procedures rely on a commercial database. Bioinformatic tools to dereplicate MALDI-TOF spectra have been developed, but an open and resource-efficient tool to reduce the redundancy of microbial isolates is lacking. Here we develop “maldipickr” for de novo-clustering of MALDI-TOF spectra to dereplicate and select isolates, and thereby facilitate large-scale cultivation projects.

**Availability and implementation:**

The R package “maldipickr” is available through CRAN at https://doi.org/10.32614/CRAN.package.maldipickr along extensive documentation at https://clavellab.github.io/maldipickr.

## 1 Introduction

There is a renewed interest in cultivation to study microbiomes because it helps going beyond what sequencing data can provide (Tamaki 2019, Clavel *et al.* 2025). Isolates enable functional studies of known and novel taxa (Müller *et al.* 2024, Hitch *et al.* 2025), and the design of defined microbial communities that recapitulate microbial ecosystem functions for use in fundamental and applied research (van Leeuwen *et al.* 2023). Whilst it would be ideal to isolate and maintain as many bacterial strains as possible from a given sample to obtain a comprehensive view of cultured functionality, it is barely feasible experimentally. The goal of capturing a large fraction of microbial diversity in culture, including novel taxa, needs to be balanced with the expected presence of clonal isolates, whose further processing is costly. Despite increasingly efficient workflows for genome-based identification, sequencing every isolate is also costly and poses problems for further processing of the cultures, which requires fast identification. Therefore, cost-effective methods to efficiently reduce the taxonomic redundancy in bacterial cultures from microbiomes are needed.

Protein fingerprinting of microbial isolates by MALDI-TOF mass spectrometry is commonly used for fast and cheap culture identification down to the species level (Rocca *et al.* 2025). In standard workflows, spectra are matched against commercially available databases (e.g. Bruker Biotyper), and isolates are assigned a taxonomic identity based on score thresholds. However, this process primarily supports identification, not large-scale dereplication or automated grouping of isolates and is limited to the taxa included in the commercially available database (Huschek and Witzel 2019). In routine workflows, grouping large numbers of isolates based on taxonomic identity requires manual export of identification results, filtering by score thresholds, and sorting or merging entries. This process becomes increasingly labor-intensive in high-throughput isolation campaigns, particularly when database coverage is incomplete. Plug-ins, to improve database coverage, are generated by the research community, but only for specific taxa or environments: Clostridia (Asare *et al.* 2023), important pathogens (Lasch *et al.* 2025), human gut (Plomp *et al.* 2024) or marine *Vibrio* species (Moussa *et al.* 2021). In addition, tools have been developed for database-independent identification of bacteria, using raw spectra of *Klebsiella* (Bridel *et al.* 2021) or *Bacillus* species (Uvarova *et al.* 2023), processed spectra (Sekiguchi *et al.* 2023) or peaks values (da Silva *et al.* 2023). However, these tools either cannot run from the acquired raw spectra, fail to provide code, data, or documentations, are not maintained, or are restricted to selected group of species.

All the aforementioned approaches require updates and do not perform well if the microorganisms being studied lack representatives in the reference database (Huschek and Witzel 2019). *De novo* approaches that cluster similar spectra are an alternative (Strejcek *et al.* 2018, Dumolin *et al.* 2019, Clark et al., 2022, Krull *et al.* 2026), if they can be easily implemented during high-throughput isolation campaigns. This work aimed to fill a gap in microbial bioinformatics by developing an open-source and convenient tool to import MALDI-TOF data, dereplicate and select isolates either from taxonomic identification or the acquired spectra, thereby facilitating microbial isolation processes.

## 2 Implementation

We established an R package to process and dereplicate MALDI-TOF spectra to select bacterial isolates. We used the R package fusen to develop maldipickr to focus on documenting and testing all functions early on as per good scientific software practice. This resulted in a robust package available on CRAN and thus easy to install on multiple platforms (Windows, Mac, Linux).

Microbiologists are presented with two paths depending on the type of data they want to use to dereplicate their isolates: spectra data or taxonomic identification reports (Fig. S1, available as supplementary material at *Bioinformatics Advances* online). Dereplication using spectra data starts with the import of the MALDI-TOF data and their processing based on methods by MALDIquant (Gibb and Strimmer 2012) and Strejcek *et al.* (2018). The raw spectra from Bruker Biotyper are first variance stabilized using square-root transformation of the signal. Spectra masses are trimmed to 4–10 kDa as they were deemed most determinant by Strejcek *et al.* (2018) and subsequently smoothed using the Savitzky-Golay method and a half window size of 20. Spectra are further preprocessed with a baseline correction using the SNIP algorithm and normalized by Total Ion Current, until peaks are detected using the SuperSmoother procedure and with a signal-to-noise ratio above 3. Peaks with negative signal-to-noise ratio are further discarded. Peak values and intensities from different spectra are merged to obtain a matrix, from which a similarity matrix between spectra is computed. By default, as in the Strejcek *et al.* (2018) procedure, the intensity values for spectra with missing peaks are interpolated from the processed spectra signal. Similarities are computed outside of maldipickr to accommodate various similarity metrics and lower maldipickr’s dependencies number. We use the cosine similarity efficiently computed with coop as in Strejcek *et al.* (2018). Hierarchical clustering then groups spectra in the same cluster only if the similarity between the spectra is above (or equal to) the provided threshold. The default and recommended method is complete linkage, also known as the farthest neighbor, to ensure that the within-group minimum similarity of each cluster respects the threshold. Once clusters are generated, representative spectra per cluster can be selected optionally using external metadata to complete the dereplication (e.g. growth parameters of the isolates). User-flagged spectra or entire clusters containing flagged spectra can optionally be masked from the selection process to accommodate more complex design.

Dereplication using taxonomic identification data starts with importing and tidying identification reports, which is made easy in maldipickr with dedicated formatting functions. Next, the user chooses a threshold for the log-score, above which the identifications are kept to form clusters of spectra with the same taxonomy. Otherwise, spectra are uniquely labeled to form their own cluster. Once clusters are generated, representative spectra per cluster can be selected similarly to the dereplication using spectra data.

## 3 Dereplication with maldipickr

We tested the features of maldipickr based on a cultivation scenario where a microbiologist has 80 isolates and wants to keep only non-redundant bacteria. Here the 80 isolates were selected from in-house collections (Wylensek *et al.* 2020, Hitch *et al.* 2025), including 8 replicates of each 9 known and 1 novel species, now described taxonomically (Hitch *et al.* 2025). Together these species belonged to 3 phyla: 7 *Bacillota*, 2 *Bacteroidota*, and 1 *Pseudomonadota* (Table S1, available as supplementary material at *Bioinformatics Advances* online). We cultured the strains and acquired MALDI-TOF spectra using a Bruker Biotyper Sirius system (Bruker Daltonics), as indicated in the Supplementary Methods, available as supplementary material at *Bioinformatics Advances* online. We then used different approaches to reduce the expected redundancy of the isolates.

**Figure 1:**
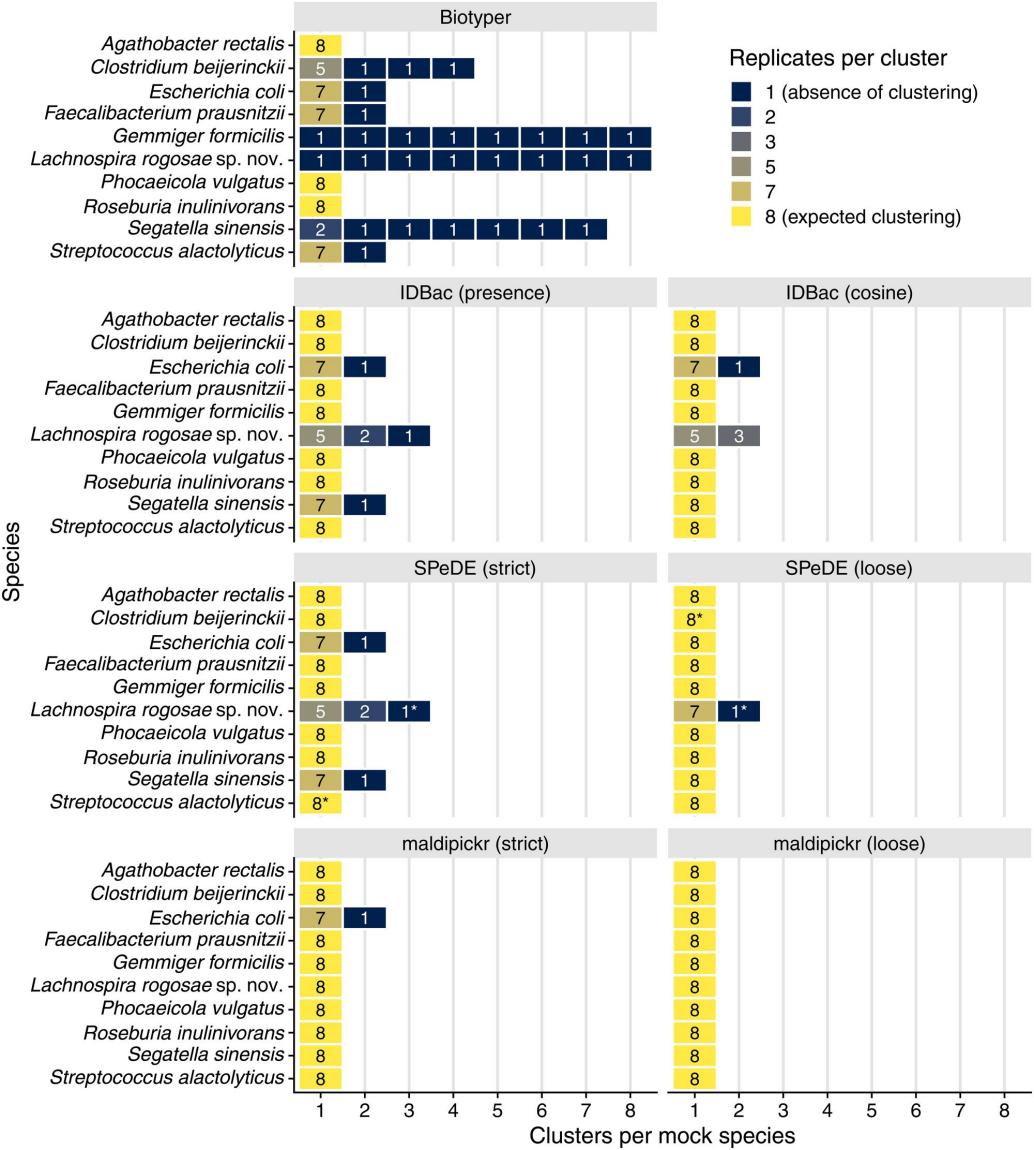
Comparison of dereplication approaches using a mock dataset of 80 MALDI-TOF spectra (8 technical replicates × 10 species). We assessed 4 dereplication approaches: identification-based using the Biotyper database (above log-score of 2), IDBac with cosine distance with selected peaks ignored (presence) or as is (cosine), SPeDE with default (strict: 50) or species-level correlation threshold (loose: 20), or maldipickr with two similarity cosine thresholds (loose: 0.79; strict: 0.92). The number of replicates per cluster for each species is indicated by color-coded bars and numbers. It ranges from 1 replicate per cluster (absence of clustering) to 8 replicates per cluster (expected clustering). Clusters with a star indicate clusters where spectra from different mock species were lumped into the same clusters by the dereplication approach. *Gemmiger formicilis* and *Lachnospira rogosae* sp. nov were absent from the Biotyper database at the time of the analysis (28 March 2023).

First, we used the identification reports from the Biotyper to dereplicate the isolates, with a streamlined data import and cleaning by maldipickr. Species identified confidently (log-score ≥ 2) were clustered with one representative to be picked, whilst unidentified spectra were all picked suggesting they may not be represented in the database. Using this method, users would have kept 36 isolates for further analysis (Table S2, available as supplementary material at *Bioinformatics Advances* online), with many species split across clusters (Fig. 1). Alternatively, we used IDBac (Krull *et al.* 2026) and SPeDE (Dumolin *et al.* 2019) to dereplicate the isolates. Whilst spectra processing was done outside of the package, we respectively imported the similarity matrix and the cluster results in maldipickr (Supplementary Methods, available as supplementary material at *Bioinformatics Advances* online). From 80 isolates, users would have kept 14 isolates using IDBac with default setting (presence). Switching to cosine distance with IDBac (cosine) would correctly lump two clusters and result in 12 isolates to be kept (Fig. 1 and Table S2, available as supplementary material at *Bioinformatics Advances* online). Using SPeDE, users would have kept 13 isolates (Table S2, available as supplementary material at *Bioinformatics Advances* online) with the default correlation threshold (strict: 50). This number decreased to 10 isolates (Fig. 1 and Table S2, available as supplementary material at *Bioinformatics Advances* online) using the species-level threshold (loose: 20). Finally, we used maldipickr to dereplicate 80 isolates (Fig. S2, available as supplementary material at *Bioinformatics Advances* online), following the implementation described above (Strejcek *et al.* 2018). Using this method, users would have kept the correct number of 10 isolates when using a loose similarity threshold (loose: 0.79); using a stricter threshold as in Strejcek *et al.* (2018) (strict: 0.92), 11 isolates would have been kept, adding an extra cluster (Fig. 1 and Table S2, available as supplementary material at *Bioinformatics Advances* online).

Next, we used common classification metrics to assess how well the isolates were grouped into species: an adjusted Rand index (ARI) of 1 indicates all spectra are into their expected species cluster; an ARI of 0 indicates random clustering. Dereplication with maldipickr achieved the highest ARI, whilst the Biotyper identification was lowest (Table S2, available as supplementary material at *Bioinformatics Advances* online). A complementary way to evaluate the clusters is with homogeneity (low means lumping) and completeness (low means splitting). Whilst maldipickr, Biotyper and IDBac delineate “pure” clusters (homogeneity of 1), SPeDE misassigned the spectra of *Lachnospira rogosae* sp. nov twice (Fig. 1 and Table S3, available as supplementary material at *Bioinformatics Advances* online), reducing the homogeneity (Table S2, available as supplementary material at *Bioinformatics Advances* online). As expected, maldipickr (strict) decreased slightly the completeness with an extra isolate picked. As two strains (*Gemmiger formicilis* and *Lachnospira rogosae* sp. nov) were absent from the Biotyper database, we reevaluated the approaches without them (Table S2, available as supplementary material at *Bioinformatics Advances* online). Not only this deletion improved the performance of Biotyper as expected, it also highlighted that similarity-based approaches were superior to database approaches in the evaluated setting.

We then used maldipickr at scale to attempt strain-level dereplication using data from Asare *et al.* (2023). After removing spectra with low-quality and ambiguous taxonomy, we used 2078 spectra from 86 strains and 34 species, where 19 species had more than one strain. We processed the spectra with maldipickr in 2–3 minutes on a standard laptop (Supplementary Methods, available as supplementary material at *Bioinformatics Advances* online) to generate 61 and 124 clusters with the loose and strict thresholds, respectively (Table S4, available as supplementary material at *Bioinformatics Advances* online). The loose threshold recapitulated clustering at the species level (ARI value of 0.72), whilst the strict threshold provided strain-level clustering (ARI value of 0.77) (Table S4, available as supplementary material at *Bioinformatics Advances* online), although some strains still grouped together (Table S5, available as supplementary material at *Bioinformatics Advances* online).

When using similarity-based approaches like maldipickr, we emphasize that ensuring adequate linkage method is paramount, as 3–4 linkage methods created clusters with similarities below the specified threshold (Fig. S3, available as supplementary material at *Bioinformatics Advances* online). Divergent spectra with as little as 0.28 and 0.50 similarity were grouped in the same cluster when using single linkage (Fig. S3, available as supplementary material at *Bioinformatics Advances* online). Additionally, the six linkage methods produced cluster counts that differed by up to fourfold (Table S6, available as supplementary material at *Bioinformatics Advances* online). The default and recommended method in maldipickr is the complete linkage (used in Fig. 1 and Fig. S2, available as supplementary material at *Bioinformatics Advances* online) to ensure that the within-group minimum similarity of each cluster respects the threshold.

Overall, we demonstrate that maldipickr achieves good dereplication compared to other approaches whilst using a narrower and potentially less noisy mass range (4–10 kDa versus 2–20 kDa for Biotyper and SPeDE; 1.8–21 kDa for IDBac).

## 4 Conclusion and future avenues

maldipickr addresses two important needs of microbiologists during microbial isolation campaigns: fast identification of non-redundant clonal cultures and the exclusion of previously selected species. This R package was developed to offer a simple, yet efficient method that requires low computing resources in comparison to machine-learning approaches; maldipickr can run on the computer piloting the mass spectrometer, as it is easy to install on multiple operating systems, and it can then use the raw spectra directly. Finally, the approach is fast and scalable, making it suitable as a building block of high-throughput isolation platforms.

Alternatively to maldipickr, microbiologists can still use Biotyper to dereplicate known isolates (especially of clinical relevance) or make use of the good dereplication performance of SPeDE. In addition, IDBac offers an online portal for interactive dereplication, including the analysis of secondary metabolite production. External data can also be used in maldicpikr, but we could not demonstrate this feature due to space limitation, even though the R package vignette showcases nonetheless how to guide isolate picking by importing metadata (e.g. growth measurement using optical density values).

Whilst maldipickr is ready to use for modest and large isolation campaigns, we plan to develop it further as issues are already faced by the user-base (including experiments in our own group). This includes the following features, which are however beyond the scope of this article. First, instead of having to choose a path (Fig. S1, available as supplementary material at *Bioinformatics Advances* online), maldipickr could integrate taxonomic identifications in the de novo-clustering approach whether they are from the Bruker database or alternative sources, such as IDBac (Krull *et al.* 2026). Second, maldipickr could also be augmented by implementing a dashboard to orchestrate efficient dereplication directly in the wet-lab. Third, defining cut-offs is the crux and curse of similarity-based approaches, as optimal thresholds across taxa are unknown. Like the developments from Operational Taxonomy Units into Exact Sequence Variants (Callahan *et al.* 2017), we envision that maldipickr could use denoised spectra without cutoffs to dereplicate isolates at the strain level. This would require a better understanding of (1) the type of background noise generated during sample preparation by including additional technical and analytical replicates (Topić Popović *et al.* 2023), and (2) the reproducibility of an isolate fingerprint across time. Finally, maldipickr could be extended to dereplicate fungal isolates and thereby expand the scope of large-scale isolation projects from microbiomes.

## Supplementary Material

vbag171_Supplementary_Data

## Data Availability

Code and data are available at https://github.com/ClavelLab/maldipickr_manuscript and https://zenodo.org/records/15744631 to reproduce the analysis and figure, or at CodeOcean at https://doi.org/10.24433/CO.1202249.v2. Data from Asare et al 2023 was obtained from https://doi.org/10.5281/zenodo.7773644.
