# Supplementary material for: maldipickr dereplicates microbial MALDI-TOF spectra to facilitate multiplexed isolation
Source: Bioinform Adv. 2026 Jun 17;6(1):vbag171. doi: 10.1093/bioadv/vbag171 (PMC13317968; doi:10.1093/bioadv/vbag171)
Supplement: vbag171_Supplementary_Data [file vbag171_supplementary_data.zip › 2026-05-20_maldipickr_SupplementaryMaterials.docx]

**Supplementary Materials**

**Supplementary Material Legends**

**Figure S1**: Overview of the two analysis paths of maldipickr, depending on the type of data microbiologists want to use to dereplicate their isolates: taxonomic identification reports or MALDI-TOF spectra data (before any processing in Bruker MSP). Text in the circles stand for the names of maldipickr R functions.

**Figure S2**: Dendrogram of 80 spectra after processing with maldipickr. Hierarchical clustering using complete linkage was used from the cosine similarity matrix. Vertical lines show the two similarity thresholds used (loose: 0.79 in dashed line; strict: 0.92 in solid line).

**Figure S3**: Distribution of the minimum cosine similarity per cluster depending on the type of clustering linkage method. Values are shown after processing the Asare et al. (2023) data using maldipickr with 0.79 (top) and 0.92 threshold (bottom). Horizontal lines show the two expected similarity thresholds. The total number of clusters is indicated in Table S6

**Table S1**: List of the isolates used in this study, including their taxonomy, cultivation media and persistent strain identifiers.

**Table S2**: Clustering metrics for each dereplication approaches tested, including the number of clusters, the Adjusted Rand Index, homogeneity and completeness. Clustering metrics were also recalculated after removing the two isolates absent from the Biotyper database (*Gemmiger formicilis* and *Lachnospira rogosae* sp. nov).

**Table S3**: Clustering results for all spectra in each dereplication approaches tested. The cluster, to which all spectra belongs within an approach, is indicated by the membership column with a number. The species name and strain identifier, from which the spectrum was generated, is provided. The column ’to_pick’ indicates whether the spectrum (or isolate) is selected by the dereplication approach.

**Table S4:** Clustering metrics for two dereplication approaches (maldipickr 0.79 and 0.92) tested with the data from Asare et al. (2023), including the number of clusters, the Adjusted Rand Index, homogeneity and completeness. Values were calculated both at the species and strain level.

**Table S5:** Clustering results for all spectra in the two dereplication approaches (maldipickr 0.79 and 0.92) tested with the data from Asare et al. (2023). The cluster, to which all spectra belong within an approach, is indicated with a number in the membership column. The genus, species, and strain name, from which the spectrum was generated, is provided. The column ’to_pick’ indicates whether the spectrum (or isolate) was selected by the dereplication approach.

**Table S6:** Total number of clusters and singletons clusters depending on the type of clustering linkage method. Values are shown after processing the Asare et al. (2023) data using maldipickr with 0.79 and 0.92 thresholds.

**Supplementary Methods**

**Cultivation of strains**

Selected bacterial strains (Table S1) were reactivated from cryo-stocks in 9 ml of their respective cultivation media. To generate sufficient biomass for downstream analysis, 1 ml of the reactivated culture was subcultured into 9 ml of fresh medium in Hungate tubes to be incubated anaerobically (89.3% nitrogen, 6% carbon dioxide, 4.7% hydrogen) for 24 hours at 37 °C.

**Preparation for MALDI-TOF analysis**

Following cultivation, 50 µl of each bacterial culture was transferred into each of 8 individual wells of a 96-well V-bottom plate (Thermo Fisher Scientific; Cat. No. 277143). Each sample was diluted with 100 µl of sterile water and centrifuged at 6,000 g for 30 minutes using a Hettich Rotanta 460 centrifuge. The supernatant was carefully discarded, and the resulting pellet was resuspended in 20 µl of sterile water.

Next, 1 µl of resuspended sample was transferred onto a MALDI target plate. After air-drying, each spot was overlaid sequentially with 1 µl of 70% formic acid (Sigma-Aldrich; F0507) and 1 µl of HCCA matrix solution, prepared by dissolving 2.5 mg α-Cyano-4-hydroxycinnamic acid (Sigma-Aldrich; 900666) in 250 µl of organic solvent (Bruker Daltonics; 8255344). Mass spectra were acquired using a MALDI Biotyper Sirius system (Bruker Daltonics) following the manufacturer’s protocol, with an adapted accumulation method summing 800 satisfactory laser shots per spot.

**Benchmark workflow**

Raw spectra data in the form of a hierarchy of folders containing acqu files were downloaded from Zenodo (<https://zenodo.org/records/15744631>), along with the identification report in the form of a semi-colon separated csv file. The IDBac workflow was run via GNPS2 (<https://gnps2.org/workflowinput?workflowname=idbac_analysis_workflow>) after conversion of the spectra data to the mzML format using MALDIquantForeign (v0.14.1). IDBac was run with default parameters using the presence and the cosine distance. The presence distance uses the cosine distance but removes selected peak intensities to limit batch effect. The Query-Query similarity matrices were downloaded and processed with maldipickr similarly to the interactive notebook on IDBac: using average linkage and a cut-height of the dendrogram of 0.7 which translates to a similarity threshold of 0.3. To create a reproducible analysis, R packages dependencies were managed using renv (Ushey and Wickham 2023) whilst the Python dependencies of SpeDE were managed from R using uv. The benchmark of the different dereplication approaches and the analysis of data were orchestrated from R using targets (Landau 2021). Additionally, clustering results from SPeDE (Dumolin *et al.* 2019) were imported and tidied in maldipickr with dedicated formatting functions. The figure was produced in R version 4.3.1 (2023-06-16) using ggplot2 (Wickham 2016), cowplot (Wilke 2024) and ggh4x (van den Brand 2025). All the analysis were ran on a Lenovo ThinkPad P15s Gen2 laptop with 8 CPUs 11th Gen Intel(R) Core(TM) i7-1165G7 @ 2.80GHz and 32 GB (2x 16GB DDR4) using Ubuntu 20.04.6 LTS.

**References**

van den Brand T. *ggh4x: Hacks for “ggplot2.”*, 2025.

Landau WM. The targets R package: a dynamic Make-like function-oriented pipeline toolkit for reproducibility and high-performance computing. *Journal of Open Source Software* 2021;**6**:2959.

Ushey K, Wickham H. *Renv: Project Environments*., 2023.

Wickham H. *ggplot2: Elegant Graphics for Data Analysis*. Springer-Verlag New York, 2016.

Wilke CO. *cowplot: Streamlined Plot Theme and Plot Annotations for “ggplot2.”*, 2024.
