# Supplementary figures and images for: maldipickr dereplicates microbial MALDI-TOF spectra to facilitate multiplexed isolation
Source: Bioinform Adv. 2026 Jun 17;6(1):vbag171. doi: 10.1093/bioadv/vbag171 (PMC13317968; doi:10.1093/bioadv/vbag171)

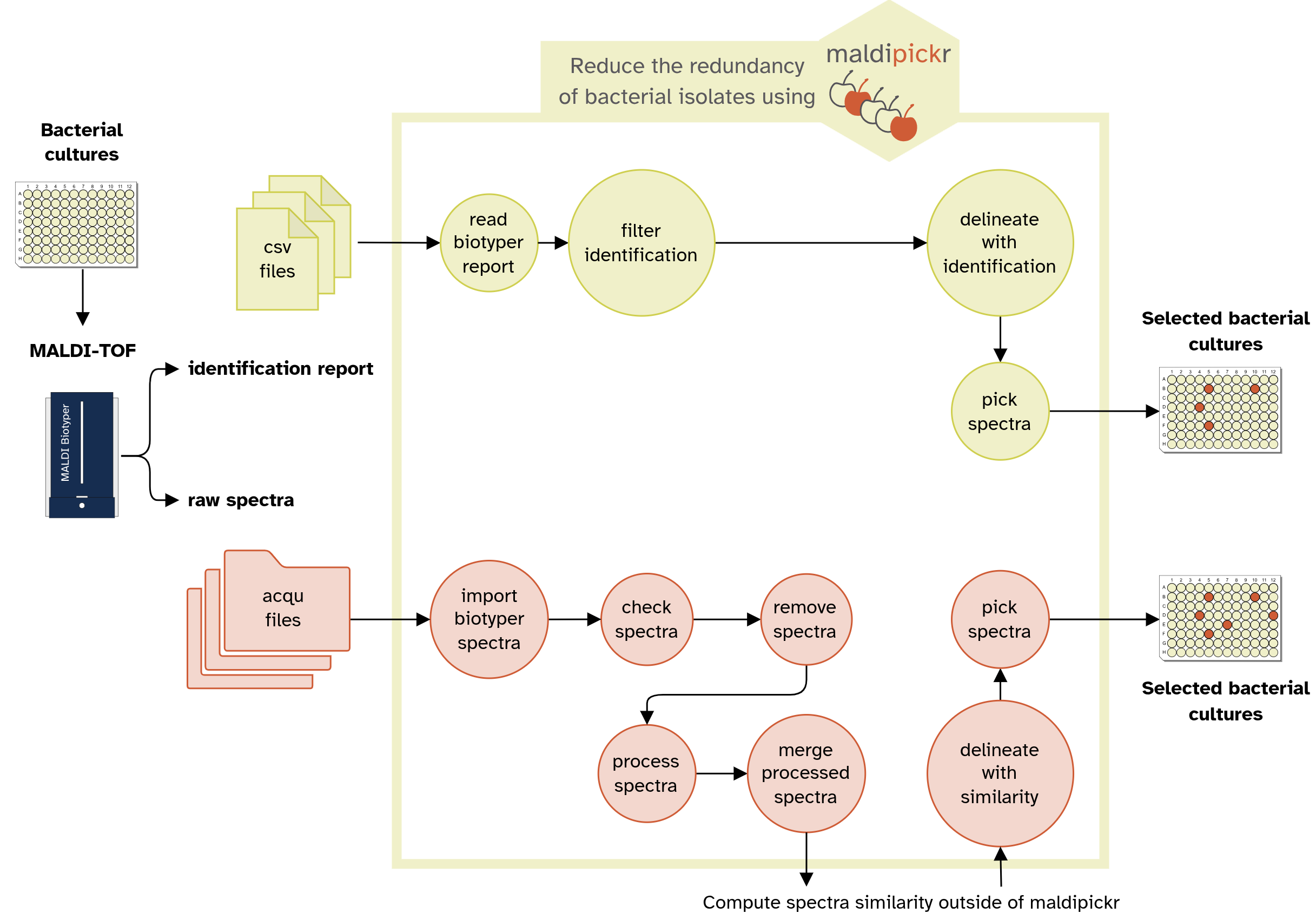

Supplement: vbag171_Supplementary_Data [file vbag171_supplementary_data.zip › FigureS1v3.png]

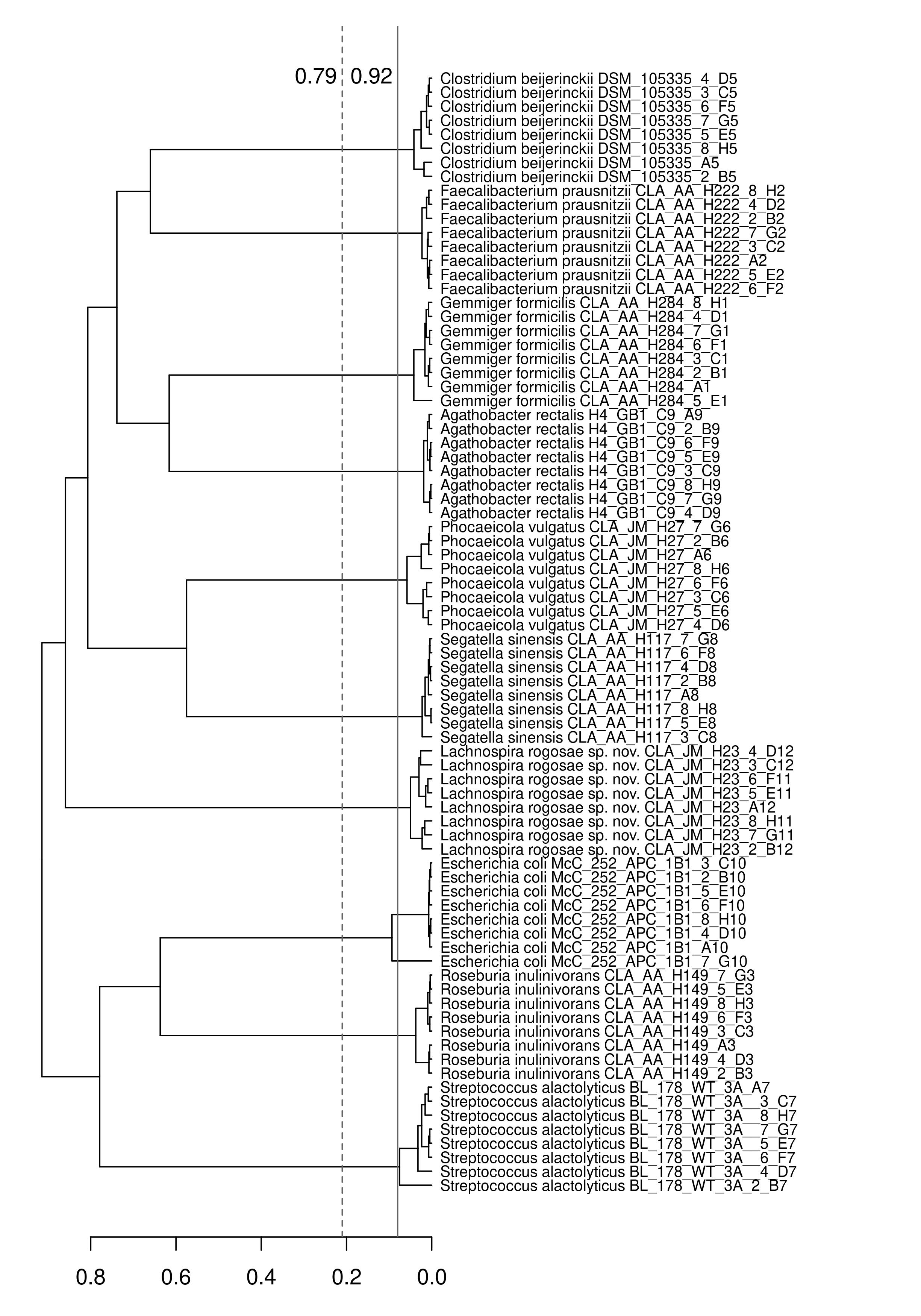

Supplement: vbag171_Supplementary_Data [file vbag171_supplementary_data.zip › FigureS2.jpg]

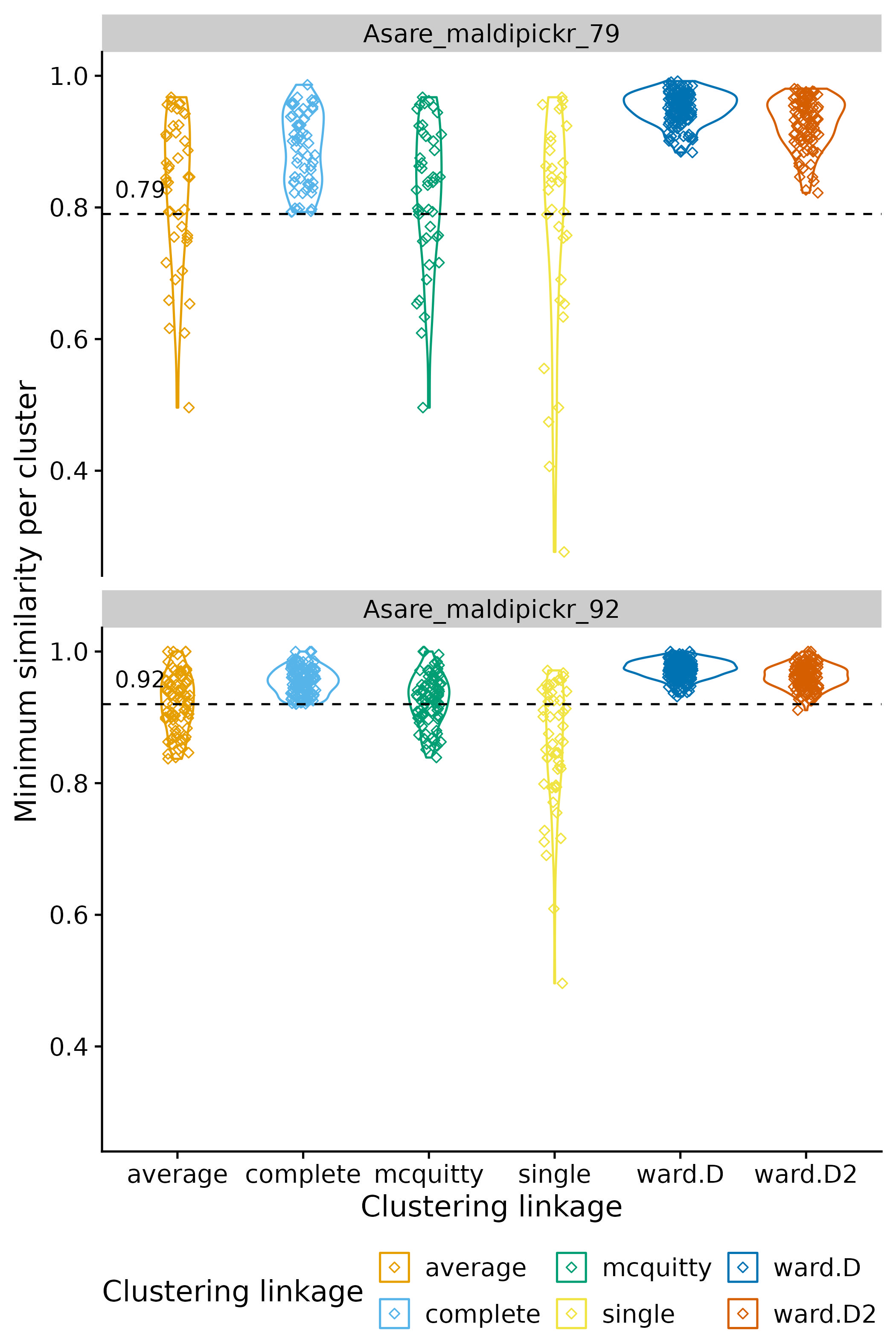

Supplement: vbag171_Supplementary_Data [file vbag171_supplementary_data.zip › FigureS3.jpg]
